# A mixed methods evaluation of a 4-week geriatrics curriculum in strengthening knowledge and comfort among orthopaedic surgery residents

**DOI:** 10.1186/s12909-021-02716-6

**Published:** 2021-05-17

**Authors:** Adrian C H Chan, Victoria Chuen, Andrew Perrella, Guillaume Limfat, Karen Ng, Vicky Chau

**Affiliations:** 1 Temerty Faculty of Medicine, University of Toronto, Toronto, Canada; 2 Department of Medicine, University of Saskatchewan College of Medicine, Saskatoon, Canada; 3 Faculty of Health Sciences, Department of Medicine, McMaster University, Hamilton, Canada; 4 Queen’s University School of Medicine, Kingston , Canada; 5 Department of Medicine, Western University Schulich School of Medicine & Dentistry, London, Canada; 6 Department of Medicine, Division of Geriatrics, Sinai Health System, Toronto, Canada

**Keywords:** Orthogeriatrics, Orthopaedic surgery, Geriatric Medicine, Postgraduate medical education, Curriculum, Evaluation, Mixed methods

## Abstract

**Background:**

In 2014, the University of Toronto Faculty of Medicine implemented a 4-week “Orthogeriatrics” rotation for orthopaedic surgery residents. We sought to assess the rotation’s impact on trainees’ knowledge, attitudes, and behaviours toward caring for older adults, and explore areas for improvement.

**Methods:**

We used a mixed methods concurrent triangulation design. The Geriatrics Clinical Decision-Making Assessment (GCDMA) and Geriatric Attitudes Scale (GAS) compared knowledge, attitudes, and behaviours between trainees who were or were not exposed to the curriculum. Rotation evaluations and semi-structured interviews with trainees and key informants explored learning experiences and the curriculum’s impact on resident physician growth and development in geriatric competencies.

**Results:**

Among trainees who completed the GCDMA (*n* = 19), those exposed to the rotation scored higher in knowledge compared to the unexposed cohort (14.4 ± 2.1 vs. 11.3 ± 2.0, *p* < 0.01). The following themes emerged from the qualitative analysis of 29 stakeholders: Increased awareness and comfort regarding geriatric medicine competencies, appreciation of the value of orthogeriatric collaboration, and suggestions for curriculum improvement.

**Conclusions:**

These results suggest that the Orthogeriatrics curriculum strengthens knowledge, behaviour, and comfort towards caring for older adults. Our study aims to inform further curriculum development and facilitate dissemination of geriatric education in surgical training programs across Canada and the world.

**Supplementary Information:**

The online version contains supplementary material available at 10.1186/s12909-021-02716-6.

## Background

Approximately 92 % of hip fractures occur in adults aged 65 years and older [1]. Compared to other orthopaedic injuries, they are more strongly associated with mortality after one year,[2, 3] postoperative delirium,[4] loss of independence,[5, 6] and prolonged mobility limitations[2].

### Models of orthogeriatric care

Orthogeriatric co-management care models were developed in the 1960’s to address these poor outcomes [7]. Briefly, orthogeriatric care involves the interdisciplinary management of elderly patients with fragility fractures, [8–11] including geriatricians and specialized allied health teams on admission [12]. Literature comparing geriatric consult models with orthogeriatric ward-based care showed the latter was associated with reduced surgical wait times, [13] postoperative falls and complications, [14, 15] hospital length of stay, [13, 16, 17] as well as short- and long-term mortality [16, 18].

Unfortunately, geriatricians in Canada remain scarce and concentrated in urban centers. Many older adults are therefore unable to receive the benefits of co-managed care [11]. In a recent survey, surgical trainees reported a lack of formal teaching and comfort involving perioperative management of older surgical patients [19].

### Geriatrics education in surgical training

A handful of surgical residency programs have begun to develop and implement formal geriatric teaching to improve trainees’ knowledge of caring for older patients. These range from didactic lectures to online discussions that seek to address learning objectives relevant to perioperative management such as delirium, pain management, falls, polypharmacy, and rehabilitation in the elderly population [20–22]. In general, knowledge and comfort in caring for older patients improved among residents who completed the course. Initiatives to formalize geriatric competencies are still ongoing, [23] yet little progress has been made in terms of establishing clear training and curricular objectives.

In 2014, the University of Toronto Department of Orthopaedic Surgery launched a 4-week Orthogeriatrics curriculum for postgraduate year 1 (PGY1) orthopaedic surgery residents throughout the year. Curricular components comprised: geriatric preoperative assessment, perioperative management of frail older adults with multimorbidity, inpatient and clinic-based geriatric assessments, and formal geriatrics educational seminars (Table 1).

**Table 1:** Orthogeriatrics curriculum components

Activity	Description
Clinical	Inpatient 2 weeks perioperative assessments: • Orthopaedic elderly patients and management of medically frail, complex older adults 2 weeks inpatient geriatric consultation: • Comprehensive geriatric assessments on the Orthogeriatrics and surgical services Outpatient 1 day Perioperative Assessment Clinic ½ day Falls Prevention Clinic ½ day Geriatric Day Hospital Clinic ½ day Geriatric Medicine Clinic
Educational Content	Perioperative Teaching Rounds (Perioperative Risk Assessment, Evidence Based Medicine, Perioperative Management e.g. Anticoagulation) Geriatric Giant Seminars (Dementia, Delirium, Falls, Incontinence, Constipation, Polypharmacy) Allied Health Seminars (Gait aids, Community Support Services, Capacity, Wound care)
Evaluation	In-Training Evaluation Reports (ITERs) 360 feedback Exit interviews

This study sought to answer the following research questions:

1. What is the impact of the Orthogeriatrics curriculum on the residents’ knowledge and attitudes pertaining to the management of elderly patients?
2. What are the areas of improvement within the current curriculum that will allow for further development?

## Methodology

**Fig. 1:**
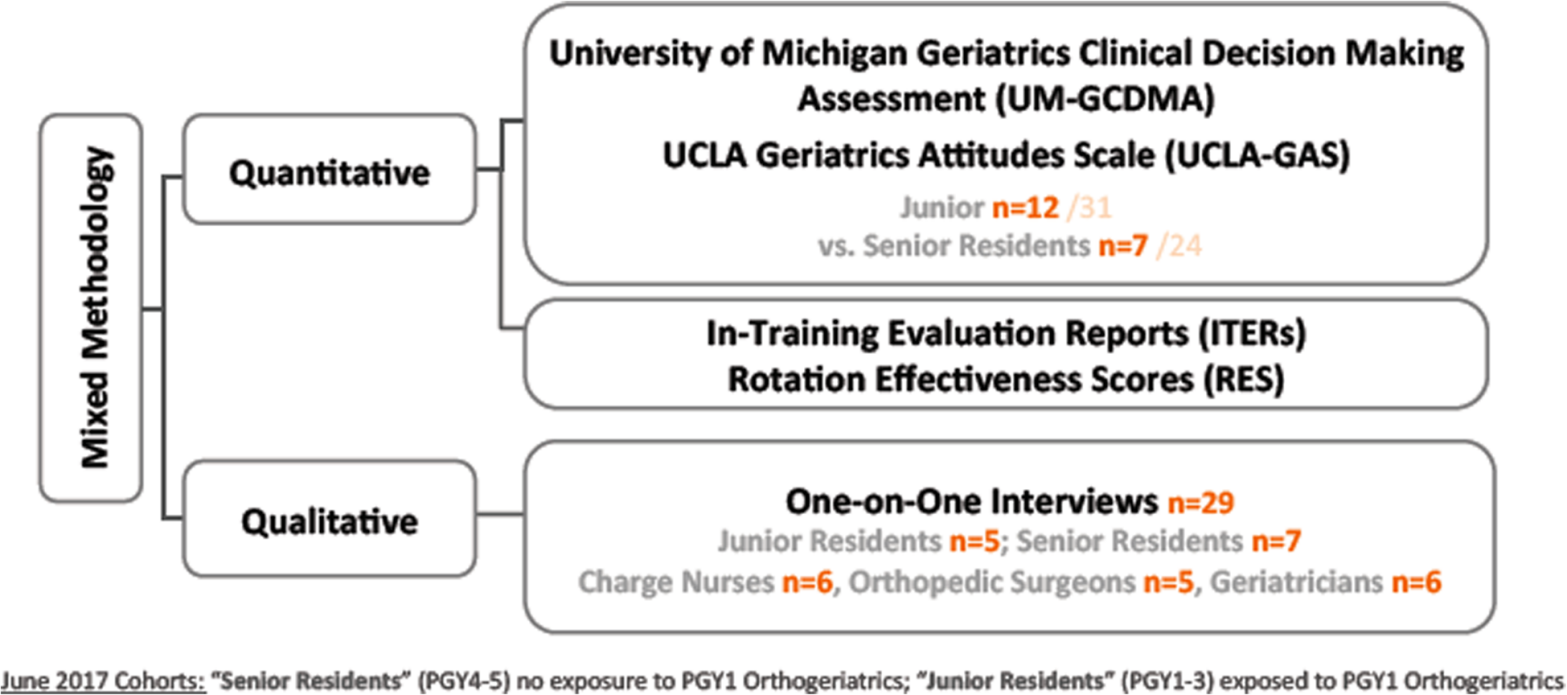
Summary of the data collected using a mixed methods triangulation design

Our mixed methods concurrent triangulation design involved the simultaneous collection and analysis of quantitative and qualitative data (Fig. 1). We assessed residents using validated geriatric knowledge and attitude evaluations and converged these findings during data analysis with themes that emerged from semi-structured interviews with the residents and key informants. The rationale for this approach was that corroboration between the two types of data would strengthen the validity of the program evaluation, and more robustly facilitate curriculum improvement.

### Study participants

We collected data using a convenience sampling method from three participant groups: junior residents (PGY1-3) who had completed the rotation, senior residents (PGY4-5) who had not completed the rotation, and key informants (nurses, orthopaedic surgeons, and geriatricians) who worked closely with the residents.

### Quantitative data

Junior and senior orthopaedic residents completed the University of Michigan Geriatrics Clinical Decision-Making Assessment (GCDMA) and the University of California Los Angeles Geriatric Attitudes Scale (GAS) in June 2017. The GCDMA is a validated 20-item multiple-choice test which emphasizes inpatient care and common geriatric syndromes designed for medical and surgical residents [24]. The GAS is a 14-item Likert questionnaire developed to assess healthcare providers’ attitudes towards caring for older patients [25, 26].

We also collected formal evaluations (In-Training Evaluation Reports [ITER] filled out by staff and Rotation Effectiveness Scores [RES] filled out by the junior residents) during the completion of their Orthogeriatrics rotation (Supplementary Files – ITER, RES).

### Qualitative data

 A research assistant [AC] conducted formal semi-structured interviews with all three study participant groups (Supplementary File – Interview Questionnaires). Junior and senior trainees were asked about their opinions on geriatric education in the orthopaedic surgery residency program, as well as their feelings of preparedness in caring for the elderly. Likewise, key informants were asked about any perceived changes in the attitudes and practices of orthopaedic surgery residents as a result of the Orthogeriatrics rotation.

### Data analysis

An independent-samples t-test was used to quantitatively compare the mean scores of knowledge and attitudes between junior and senior residents. Interviews were audio-recorded, transcribed verbatim, and organized using NVivo Software (Version 11.0). The data were qualitatively analyzed using grounded theory to develop a framework describing the Orthogeriatrics learning experience, its perceived impact on clinical competencies and attitudes towards caring for older adults, and areas for improved curricular performance [27]. A research assistant [AC] and PIs [VC, KN] independently read the transcripts and met periodically to refine the coding structure. This process continued until consensus was met and the coding structure was deemed stable. The research team then coded all transcripts and examined key themes that emerged. Qualitative analysis was performed concurrently with data collection to confirm that the interviews captured the information that we anticipated and concluded when theoretical saturation was achieved.

## Results

### Quantitative data

12 of 31 junior residents (39 %) and 7 of 24 (29 %) senior residents completed the GCDMA and GAS (see Table 2). We suspect that the low number of respondents was due to the voluntary nature of these assessments and that, although an effort was made to give residents protected time to complete them during their Academic Half Day (i.e., weekly teaching sessions), perhaps they had clinical duties that prevented them from participating.

**Table 2:** Comparison of junior vs. senior resident scores on geriatric knowledge and attitudes

	Number of Participants	GCDMA (mean ± SD) /20	GAS (mean ± SD) /70
Junior Residents	12	**14.4** ± 2.1	51.8 ± 4.9
Senior Residents	7	**11.3** ± 2.0	51.7 ± 8.1
*p*-value		0.009*	0.979

*GCDMA* Geriatric Clinical Decision-Making Assessment; *GAS *Geriatric Attitudes Scale

### Knowledge and attitudes

Junior residents scored statistically significantly higher on the GCDMA when compared to senior residents (14.4 ± 2.1 vs. 11.3 ± 2.0, *p* = 0.009). No difference was found between the two groups on the GAS (51.8 ± 4.9 vs. 51.7 ± 8.1, *p* = 0.979).

### Rotation effectiveness scores

Residents provided the following evaluations for the Orthogeriatrics rotation: 3.95 (2014–2015), 3.93 (2015–2016), and 4.25 (2016–2017). The scores, which are averaged out of 5, assess the curriculum’s overall organization, educational design, learning supports, climate, experience, and facilities (Supplementary File - RES).

### Qualitative data

Interviews were conducted with junior residents (JR; *n* = 5), senior residents (SR; *n* = 7), charge nurses (CN; *n* = 6), orthopaedic surgeons (OS; *n* = 5), and geriatricians (G; *n* = 6). The emerging themes are outlined below and in Table 3:

**Table 3:** Emerging themes from qualitative analysis of semi-structured interviews

1. Awareness and comfort in geriatric medicine competencies • A sense of increased comfort in various competencies involved in geriatric assessment and management, noted by both residents and nursing staff
2. Geriatric competencies strengthened by the curriculum: • Sensitization to Holistic Care and Medical Complexity • Communication with Older Adults • Collaborative Relationships
3. Improving the Orthogeriatrics curriculum • Suggestions for improvements in curricular design, such as pre-operative management of elective cases, psychosocial dynamics with caregivers and families, and different pain management modalities

### Awareness and comfort in geriatric competencies

Junior residents felt comfortable in their geriatric medicine competencies, particularly in the initial management of the geriatric giants. They were also able to extrapolate their knowledge to work through issues during a patient’s hospital stay.

“I am comfortable in taking the first steps in getting them (older adults) optimized.” (JR4).

“It really makes you feel more confident moving forward in terms of how to deal with these ward issues.” (JR3).

In contrast, senior residents had mixed comfort in geriatric medicine competencies. Some felt their knowledge and skills were “probably a little limited” (SR5) and “would not be sufficient to provide a standard of care.” (SR6) Nursing staff also commented that senior residents were sometimes “not sure how to proceed because of all the medical complications.” (CN1).

Geriatric competencies strengthened by the curriculum

### Geriatric competencies strengthened by the curriculum

Among the geriatric competencies, three sub-competencies appeared to be positively affected by the Orthogeriatrics curriculum.

1. *Sensitization to Holistic Care* & Medical Complexity: Junior residents recognized the importance of comprehensive care, given the medical complexity of older surgical patients. As residents “learned to assess older adults more holistically”, they began to see beyond the “mechanical” and “surgical” aspects of orthopaedic care (JR6). They also spoke of transferring their knowledge acquired from the geriatrics rotation to their surgical training:

“It’s not just the surgery, it’s the patient as a natural person that has […] dementia or social problems. And now, when you get back into more surgical training, it’s actually in your mind.” (JR5).

2. *Communicating with Older Adults*: Junior residents also appeared to interact with older adults with improved bedside manner. Role-modelling appeared to play a significant role in developing these communications skills.

“Seeing how much time the internists and geriatricians spend really validates the fact that I might spend an extra bit of my time on the overnight call talking with them.” (JR2).

Since the Orthogeriatrics curriculum, charge nurses observed “more conversation” (CN5) and “one-to-one patient time spent from the MDs.” (CN6).

3. *Collaborative Relationships*: Understanding the role of the geriatrician and allied health professionals involved in patient care appeared to strengthen interprofessional appreciation among junior residents.

“It (Orthogeriatrics rotation) gives you more tools to know when to refer … it gave me the potential to appreciate how important it is, and what to expect in a referral, and how to better prepare a patient before the geriatric team sees a patient.” (JR5).

### Improving the orthogeriatrics curriculum

The Orthogeriatrics rotation focused on managing older adults with emergent hip fractures. One suggestion was to teach about “perioperative assessments” (G2) for patients undergoing elective procedures.

“There should be an emphasis more on the pre-op assessment. So pre-ops for […] all the elective cases - the elective knee replacements, hip replacements - they probably need more teaching and experience around that.” (G3).

In addition, disposition planning within complex psychosocial situations “to help deal with caregivers and family” (OS4) could be integrated more into the curriculum.

“… whatever setting orthopaedic residents are going to end up practicing in, that they have collaborative relationships […] and make sure that their communication is open with the families and caregivers.” (OS1).

## Discussion

This research aims to further the current development of orthopaedic/geriatric care models and competences both in training and in practice [28–30].

Within clinical guidelines worldwide, orthogeriatric management for patients with a fragility fracture is the expected standard of care [31]. However, as echoed by our participants, a residents’ scope of practice upon completion of their training remains highly variable, and resource limitations may limit a surgeon’s access to a collaborative care model. Indeed, there were mixed opinions among senior orthopaedic residents who had not completed the Orthogeriatrics curriculum on whether they felt comfortable providing care to medically complex older patients. These sentiments are reflected in the literature among surgical faculty and residents, which further highlight the need for further geriatric training in medication, comorbidity, and delirium management [32].

Overall, the enhanced comfort and confidence in decision-making around older adult patients was noted by residents, surgeons, and charge nurses – especially as these were felt to be new traits among junior resident cohorts. The ability to transfer their learning from the curriculum to other services/rotations was of particular interest, as a prime concern was that such experiences in the PGY1 year would become lost amidst their rigorous surgical training. However, it appeared that the comfort with older adults the junior residents acquired has permeated longitudinally during their training thus far.

### Growth in knowledge

Most striking for us was the apparent knowledge difference in geriatric competencies between junior and senior residents, as evidenced by the GCDMA scores. Although multiple competing factors could account for this difference (e.g. a focus on licensing examinations, operative skill, and independent practice during senior residency years), it was further corroborated by input from key informants that the junior cohort of residents appeared stronger in managing complex medical issues in the older adult patient.

Geriatric knowledge assessment among surgical residents appears to be a relatively new phenomenon. Acknowledging the difficulty in drawing comparisons given the paucity of studies using standardized scales, our junior resident mean score of 72 % on the GCDMA is similar to that of geriatric fellows and senior internal medicine residents *after* their completion of a geriatrics-palliative care rotation [26, 33]. A Dutch study designed a 6-week online course for faculty and residents involved in perioperative management of frail older patients [20]. Following the completion of these modules, the investigators noted an increase in knowledge and confidence scores among its participants. However, a limitation of this study is that it lacked a control group to compare with those who completed the online curriculum. Nonetheless, it may provide a reasonable alternative that is less resource-intensive and easier to implement across surgical residency programs.

### Growth in comfort

 Our interviews reinforced the positive impact of the curriculum on comfort in geriatric medicine competencies, and more specifically, the residents’ appreciation for holistic and multidisciplinary care, as well as optimization of collaborative relationships and enhanced communication with older adults.

Another evaluation of a geriatric curriculum that used 16 h-long didactic sessions to teach general surgery residents noted increased comfort in accessing community resources and multidisciplinary care to manage issues such as postoperative delirium and acute renal failure [21]. However, this study lacked objective testing in terms of knowledge base and therefore could not comment on whether there was a simultaneous improvement in the residents’ understanding of patient management. While we and others did not find an objective impact on attitudes towards older adults on the GAS, our qualitative analysis demonstrated an overall embrace for the Orthogeriatrics curriculum from program stakeholders [26].

One should not discount the effect of role-modelling on geriatrics services in achieving holistic care. Several residents noted the opportunity to model their behaviour and patient communication based on their geriatric inpatient or perioperative hospitalist preceptors, as if being given permission to spend more time investigating a patient’s concerns. Although we postulate that rarely in a surgeon’s training would they be afforded time for lengthy consults, we believe that exposure to the culture of inpatient and outpatient geriatric care is what facilitated an appreciation for geriatric models of care, and sensitization to the older adult population.

That all participant groups applauded the necessity and applicability of Orthogeriatrics in residency training is a testament to both the growing complexity of patient care as well as gratification in being able to deliver such care.

### Sustainability of geriatrics curricula

Several barriers in the design and implementation of a geriatric curriculum for residents were identified in the literature, [34] including limited geriatric faculty and services, an uncompromising curriculum, and lack of faculty or resident interest. Our educational program is sustained through existing orthogeriatric care models and geriatric clinical services, which fortunately have required minimal additional resources or administrative support.

Nonetheless, we discovered that the structure and content of our curriculum requires further improvement. With regards to curriculum structure, further discussions may help to optimize the balance between hospitalist and inpatient geriatric services that residents receive. There is also a need to broaden our clinical experiences to include topics like perioperative assessments of additional orthopaedic disorders and pain modalities in the perioperative setting. Furthermore, an understanding of discharge planning, especially in a post-fall hospitalization, is a critical aspect to resident training. Understandably so, as patients discharged to nursing homes have been shown to have higher readmission rates and prolonged lengths of stay [35, 36]. Current co-management orthogeriatric care models pay little emphasis to resident or surgeon education on discharge planning, and thus addressing this gap would be instrumental in improving our curriculum.

### Study limitations

This study had several limitations from a research methodology perspective. First, it was challenging to recruit an adequate number of orthopaedic surgery residents. As such, the statistical comparison of geriatrics knowledge and attitudes between cohorts may not be statistically robust. Secondly, the University of Michigan GCDMA as an assessment of geriatric knowledge has been criticized for being outdated and concentrated on inpatient medicine [37, 38]. However, we employed this tool as an indicator of the knowledge growth generated by our curriculum, rather than reliance on a final numerical score. Finally, although our study utilized a validated test to evaluate the effect of a curriculum on resident knowledge, it did not explore its impact on clinically significant outcomes (e.g. adverse patient events, such postoperative delirium, lengths-of-stay, or hospital readmissions).

## Conclusions

Our comprehensive evaluation demonstrates that residents have increased knowledge and comfort with managing geriatric issues on the wards following the completion of the 4-week Orthogeriatrics curriculum. Future steps for this curriculum include integrating the suggestions identified in our evaluation study and expanding geriatrics education programs to other surgical residency programs. We hope that our pilot work may serve as a model to other medical institutions to ultimately build capacity for stronger surgical training towards providing care for older adult patients.

## Supplementary information

Additional file 1.   ITER. Clean version of Orthogeriatrics ITER.

Additional file 2.  RES. Clean version of Orthogeriatrics RES.

Additional file 3.  Interview Questionnaires. List of interview questions for study participants.

## Data Availability

The datasets used and analyzed during the current study are available from the corresponding author on reasonable request.

## Abbreviations

CN: Charge nurse
G: Geriatrician
GAS: Geriatric attitudes scale
GCDMA: Geriatrics clinical decision-making assessment
ITER: In-training evaluation report
JR: Junior resident
OS: Orthopaedic surgeon
PGY: Postgraduate year
RES: Rotation effectiveness score
SD: Standard deviation
SR: Senior resident
