# Supplementary material for: A mixed methods evaluation of a 4-week geriatrics curriculum in strengthening knowledge and comfort among orthopaedic surgery residents
Source: BMC Med Educ. 2021 May 17;21:283. doi: 10.1186/s12909-021-02716-6 (PMC8130312; doi:10.1186/s12909-021-02716-6)
Supplement: Supplementary file 1 — ITER. Clean version of Orthogeriatrics ITER. [file 12909_2021_2716_MOESM1_ESM.pdf]

Module 4_Medical consult_ITER

* Required

Toronto Orthopaedics
Leadership. Innovation. Impact.

1.) Name of Resident *

Choose

If Resident name is not listed, please list below

Your answer

2.) PGY Level *

Choose

3.) Rotation Start Date

Date

yyyy-mm-dd

4.) Rotation End Date

Date

yyyy-mm-dd

5.) Evaluator

Choose

If Evaluator name is not listed, please submit

Your answer

Descriptors For Ratings

1<br>Fails to Meet Essential Competencies	2	3<br>Meets Essential Competencies	4	5<br>Demonstrates Enhanced Competencies	6<br>U/A
• Below the minimally acceptable level for competence in the specified practice context (i.e. patient population, learning environment, practice setting).<br>• Does not know limits and/or when to ask for assistance when needed<br>• Unable to manage usual volume of work in timely manner<br>• Does not improve or respond to feedback		• Demonstrates a solid understanding of the issues, interpretation of problems and basic implementation of solutions(s)<br>• Handles common or straightforward situations and presentations competently<br>• Does what is expected in a timely way<br>• Knows and works within competence limits<br>• Generally responds to feedback		• Demonstrates ease and efficiency in handling common straightforward as well as increasingly complex situations and presentations<br>• Demonstrated excellence in his or her understanding of the issues, ability to interpret problems and implement solutions<br>• Exceeds the benchmark for competent performance<br>• Anticipates and develops areas needing refinement and strives for continuous improvement	• Unable to Assess

For this MODULE ITER, a 3 is required for successful completion

MEDICAL EXPERT *

Demonstrates diagnostic and therapeutic skills for effective patient care; applies relevant information and therapeutic options to clinical practice; demonstrates effective consultation services with respect to patient care and education; recognizes personal limits of expertise.

	1 - Fails to meet essential competencies	2	3 - Meets essential competencies	4	5 - Demonstrates enhanced competencies
1. Demonstrates and applies perioperative risk assessment tools and management strategies for anaesthetic risks, cardiopulmonary risk, delirium risk and malnutrition (i.e. demonstrates an understanding of investigations and identifies modifiable risk factors to optimize patients prior to surgery).	○	○	○	○	○
2. Demonstrates an understanding of limitations of preoperative screening tools, anticipates need for further risk stratification/screening tools.	○	○	○	○	○
3. Conducts appropriate assessment and appropriate management of issues in the preoperative and perioperative setting, including cardiac valvular disease, anticoagulation, chronic renal disease, chronic liver disease,	○	○	○	○	○

diabetes, nutrition, wounds and pain; independently provides clear plan of management of all issues.

4. Demonstrates an understanding of physiology of aging of specific organ systems and considerations in perioperative management.

5. Demonstrates an approach to the assessment, evaluation and management of the geriatric patient with regards to key issues, including delirium, dementia, depression; falls and bone health; frailty; polypharmacy and adverse drug events; urinary incontinence; and constipation (i.e. presents holistic and comprehensive management plan for the majority of the key issues).

COMMUNICATOR *

	1 - Fails to meet essential competencies	2	3 - Meets essential competencies	4	5 - Demonstrates enhanced competencies	6 - U/A
1. Develops care plan with patients and families.	○	○	○	○	○	○
2. Dialogues with patients and their families exploring philosophy of care and goals of care (i.e. facilitation of discussion surrounding philosophy of care, and clarification of issues as appropriate).	○	○	○	○	○	○

COLLABORATOR: *

Consults and collaborates effectively

	1 - Fails to meet essential competencies	2	3 - Meets essential competencies	4	5 - Demonstrates enhanced competencies	6 - U/A
1. Engages with other care providers from the community (i.e. facilitates smooth transitions into and out of the acute care setting for each patient).	○	○	○	○	○	○
2. Engages with interdisciplinary team members (i.e. attends and participates at interdisciplinary rounds, planning of patient dispositions, etc.)	○	○	○	○	○	○
3. Involves other health care professionals in developing a care plan	○	○	○	○	○	○
4. Communicates with interdisciplinary team members and care providers in the community	○	○	○	○	○	○

through dictations, consultation notes, discharge summaries, and ongoing communication between team members.

LEADER *

	1 - Fails to meet essential competencies	2	3 - Meets essential competencies	4	5 - Demonstrates enhanced competencies	6 - U/A
1. Demonstrates an understanding of transitions in care and makes arrangements between interdisciplinary team members to facilitate safe transitions as appropriate; recommends the most appropriate options for rehabilitation (slow-stream vs. regular stream, nursing home, etc.)	○	○	○	○	○	○
2. Makes cost effective use of health care resources based on sound judgment, balancing resources to maximize benefits to all patients	○	○	○	○	○	○
3. Demonstrates awareness of and/or participates in Quality	○	○	○	○	○	○

Improvement and Patient Safety initiatives to improve the care of the older adult undergoing orthopaedic procedures.

HEALTH ADVOCATE: *

	1 - Fails to meet essential competencies	2	3 - Meets essential competencies	4	5 - Demonstrates enhanced competencies	6 - U/A
1. Manages common barriers to care faced by the geriatric population across the healthcare spectrum (from inpatient, to outpatient to rehabilitation settings).	○	○	○	○	○	○

SCHOLAR *

	1 - Fails to meet essential competencies	2	3 - Meets essential competencies	4	5 - Demonstrates enhanced competencies	6 - U/A
1. Critically appraises basic evidence behind perioperative practices (i.e., cardiac risk assessment, blood transfusion thresholds, etc.).	○	○	○	○	○	○
2. Applies evidence to guide clinical management of common issues in perioperative management of geriatric orthopaedic patients.	○	○	○	○	○	○

PROFESSIONAL *

	1 - Fails to meet essential competencies	2	3 - Meets essential competencies	4	5 - Demonstrates enhanced competencies	6 - U/A
1. Demonstrates a commitment to geriatric orthopaedic patients and the individuals involved in the patient's circle of care.	○	○	○	○	○	○
2. Manages challenging ethical situations (as appropriate, including capacity assessments, Ministry of Transportation reporting, feeding tube insertion, philosophy of care discussions, elder abuse, etc.).	○	○	○	○	○	○

OVERALL COMPETENCE *

U/A - Unable to assess

	1 - Fails to meet essential compentencies	2	3 - Meets essential competencies	4	5 - Demonstrates enhanced competencies	6 - U/A
Possesses knowledge, skills, and attitudes appropriate to level of training. Inspires confidence in patients and staff.	○	○	○	○	○	○

7.) Were educational objectives / performance discussed with the resident at the BEGINNING of the rotation? *

- [ ] Yes
- [ ] No

8.) Were educational objectives / performance discussed with the resident at the END of the rotation? *

- [ ] Yes
- [ ] No
- [ ] N/A

9.) The information for this evaluation was provided by *

- [ ] an individual
- [ ] a committee

10.) If more than one person, please indicate other Teachers/Supervisors who contributed to this evaluation

- [ ] Nurse
- [ ] Physio Therapist
- [ ] Occupational Therapist
- [ ] Other:

If more than one additional contributor, please submit.

Your answer

COMMENTS

Your answer

Evaluator Code *

Your answer

Date of Evaluation

Date

yyyy-mm-dd

Page 1 of 1 Submit

Never submit passwords through Google Forms.

This content is neither created nor endorsed by Google. Report Abuse - Terms of Service - Privacy Policy

Google Forms
