# Supplementary material for: A mixed methods evaluation of a 4-week geriatrics curriculum in strengthening knowledge and comfort among orthopaedic surgery residents
Source: BMC Med Educ. 2021 May 17;21:283. doi: 10.1186/s12909-021-02716-6 (PMC8130312; doi:10.1186/s12909-021-02716-6)
Supplement: Supplementary file 2 — RES. Clean version of Orthogeriatrics RES. [file 12909_2021_2716_MOESM2_ESM.pdf]

UNIVERSITY OF TORONTO
FACULTY OF MEDICINE

Postgraduate Medical Education

Department of Medicine, Division of General Internal Medicine
Rotation and Educational Site Evaluation (RESE)

Training Session: 2016 - 2017 Block: 03-Apr-2017 - 30-Apr-2017
Training Level: PGY ONE Rotation Service: Module 4 - Medical Com in the Surg Pat -NEW [STANDARD] [CORE]
Location: MSH-MSH [Mount Sinai Hospital]

Goals and Objectives

Note: A review of the goals and objectives for this rotation may assist you in completing this form.

About this Form

About the Rotation and Educational Site Evaluation Form:

- This form is intended to rate your overall experience on this rotation at this site.
- To assess a specific teacher, use a Resident Assessment of Teacher Effectiveness Form (RATE)
- The Department of Medicine is very interested in the learner experience and needs your input to monitor, support and improve this rotation and this site's educational effectiveness.
- Honest, constructive, professional information about the rotation at each site is an important professional obligation of learners.

How we will use the information:

- Serious issues you raise may be flagged for prompt response by the program through established processes (e.g. the Program Director will receive an alert of low RES ratings and then addresses the information in a timely and sensitive manner.)
- Teaching sites and/or rotation/site co-ordinators will receive aggregate summaries of feedback, including comments. Aggregate summaries are anonymized (i.e. not linked to an individual)
- The aggregate data is used by the PGME/Department/Division to evaluate the rotations and sites on a regular basis.

The rating scale is as follows:

1 = Unsatisfactory Experience
2 = Poor Experience
3 = Good Experience
4 = Very Good Experience
5 = Superior Experience

NOTE: 3 is a 'passing' score for this rotation and site

Form

Rotation Type: (e.g. ward, OR, ICU, clinic): ward

	Unsatisfactory 1	Poor 2	Good 3	Very Good 4	Superior 5	N/A N/A
ORGANIZATION of rotation and site (e.g. registration, orientation and scheduling)	O<br>Unsatisfactory organization of rotation and site, disorganized registration, unacceptable orientation and poor scheduling.	O	O<br>Good organization of rotation and site such as registration, orientation and scheduling.	O	O<br>Superior organization of rotation and site such as highly effective registration, superb orientation and scheduling.	O

Comments on ORGANIZATION of rotation and site:

	Unsatisfactory 1	Poor 2	Good 3	Very Good 4	Superior 5	N/A N/A
EDUCATIONAL DESIGN of rotation and site (e.g. utility of goals & objectives, effectiveness of formal learning, value of 'on the job' learning)	O<br>Unsatisfactory educational design for rotation and site such as goals &	O	O<br>Good educational design for rotation and site such as utility of goals &	O	O<br>Superior educational design for rotation and site such as utility of goals &	O

	objectives weak or not present, ineffective formal learning, limited value of 'on the job' learning.		objectives, effectiveness of formal learning, value of 'on the job' learning.		objectives, effectiveness of formal learning, exceptional value of 'on the job' learning.	

Comments on EDUCATIONAL DESIGN of rotation and site:

	Unsatisfactory 1	Poor 2	Good 3	Very Good 4	Superior 5	N/A N/A
	O	O	O	O	O	O
LEARNING SUPPORTS of rotation and site (e.g. communication, supervision, graded responsibility, feedback)	Unsatisfactory learning supports for rotation and site such as weak communication; unavailable supervision; inattentive to graded responsibility; ineffective feedback.		Good learning supports for rotation and site such as communication, supervision, assignments matches ability levels and constructive timely feedback.		Superior learning supports for rotation and site such as excellent communication, excellent supervision, highly responsive to level of skill and ability and regular and detailed coaching and feedback.	

Comments on LEARNING SUPPORTS of rotation and site:

	Unsatisfactory 1	Poor 2	Good 3	Very Good 4	Superior 5	N/A N/A
	O	O	O	O	O	O
LEARNING CLIMATE of rotation and site (e.g. respectful, collegial, collaborative inter and intra professional teams)	Unsatisfactory learning climate for rotation and site such as disrespectful, not collegial, non-collaborative inter and intra professional teams.		Learning environment for rotation and site is respectful, collegial, collaborative among inter and intra professional teams.		Superior learning climate with respectful trusting relationships, and highly collegial, strong collaborative inter and intra professional teams.	

Comments on LEARNING CLIMATE of rotation and site:

	Unsatisfactory 1	Poor 2	Good 3	Very Good 4	Superior 5	N/A N/A
	O	O	O	O	O	O
EDUCATIONAL EXPERIENCE of rotation and site (e.g. balance of work assignments to formal/informal learning opportunities; case mix)	Unsatisfactory educational experience for rotation and site such poor balance of work assignments to formal/informal learning opportunities and unresponsive to learner needs for case mix.		Good educational experience for rotation and site such as balance of work assignments to formal/informal learning opportunities and appropriate case mix for learner.		Superior educational experience for rotation and site such as excellent balance of work assignments to formal/informal learning opportunities and attentive to learner needs re: case mix.	

Comments on EDUCATIONAL EXPERIENCE of rotation and site:

	Unsatisfactory 1	Poor 2	Good 3	Very Good 4	Superior 5	N/A N/A
	O	O	O	O	O	O
FACILITIES of rotation and site (e.g., adequacy, accessibility, safety, good working environment)	Unsatisfactory facilities for rotation and site such as inadequate or poor accessibility, concern for safety and poor working environment.		Good facilities for rotation and site such as adequacy, accessibility, safety and good working environment.		Superior facilities for rotation and site such as adequacy, accessibility, strong safety protocols and culture and excellent working environment.	

Comments on FACILITIES of rotation and site:

OVERALL Rating

	Unsatisfactory Experience	Weak Experience	Good Experience	Very Good Experience	Superior Experience	N/A
	1	2	3	4	5	N/A
OVERALL Rating (NOTE: 3 is a 'passing' score for this rotation and educational site evaluation)	O<br>Significant limitations to suitability of this rotation and/or educational site.	O<br>Limitations in suitability of this rotation and/or educational site.	O<br>Solid rotation and suitable educational site.	O<br>Great rotation and educational site.	O<br>Top notch rotation and educational site.	O

Comments on Overall Rating:

Describe STRENGTHS of this rotation & site:

Actions or Areas FOR IMPROVEMENT:

OTHER Comments:

Completion Date: 30-Apr-2017
