# Supplementary material for: A mixed methods evaluation of a 4-week geriatrics curriculum in strengthening knowledge and comfort among orthopaedic surgery residents
Source: BMC Med Educ. 2021 May 17;21:283. doi: 10.1186/s12909-021-02716-6 (PMC8130312; doi:10.1186/s12909-021-02716-6)
Supplement: Supplementary file 3 — Interview Questionnaires. List of interview questions for study participants. [file 12909_2021_2716_MOESM3_ESM.docx]

**Interview Questionnaire for Junior Orthopaedic Surgery Residents**

| **THEMES** | **Junior Residents**  *(PGY1-3)* |
| --- | --- |
| **Impressions** | In PGY1, you participated in a 1 month orthogeriatrics rotation.   - **What was your experience on the orthogeriatrics rotation?**    - **Educational experience: clinical (inpatient-hospitalist vs. geriatrics, clinics), education/teaching/rounds**   - **Rotation: Design? Organization? Learning context? Support? Facilities?** - **What were your orthogeriatrics goals and objectives? Were they met? If so, how and why?** - **How did the orthogeriatrics rotation add to (and/or took away from) your surgical training?** - **What suggestions if any do you have have to improve the orthogeriatrics rotation?** |
| **Geriatric Comfort** | How comfortable do you feel managing a frail, older, complex patient? **Why do you feel that way?** |
| **Effect of the Rotation** | How did the rotation change your skills, attitudes and knowledge of the geriatric population? **Why do you feel that way?** |
| **Exposure to Geriatrics** | **What are your geriatric medicine learning experiences like outside of the orthogeriatrics rotations?**  **(e.g. other clinical rotations, clinics, academic half days, conferences)** |
| **Learning in the Rotation** | The curriculum is now 3 years old, meaning that PGY 4’s and 5’s would not have been exposed to it.   - **How important is a structured geriatrics curriculum to trainees?** - **How did the orthogeriatrics curriculum add value to your residency education?** - **Thinking back to your experiences, how could orthopaedic residents benefit from greater exposure to geriatric medicine?** |
| **Role of the Orthopaedic Surgeon** | What role do you see orthopaedic surgeons/residents in geriatric medicine outside of the OR?   - Is there room for improvement, and how should this be achieved? |

**Interview Questionnaire for Senior Orthopaedic Surgery Residents**

| **THEMES** | **Senior Residents**  *(PGY4-5)* |
| --- | --- |
| **Geriatric Comfort (Knowledge, skills, and attitude)** | How comfortable do you feel managing a frail, older, complex patient? **Why do you feel that way?**  **How do you currently feel about your geriatric knowledge and skills?**  **What are your overall thoughts about the older adult population?** |
| **Exposure to Geriatrics** | **What are your geriatric medicine learning experiences? (e.g. other clinical rotations, clinics, academic half days, conferences)**  **What additional geriatric experiences would you have wanted more or less of?** |
| **Value of Geriatrics curriculum**  **After describing the rotation** | **How important is a structured geriatrics curriculum to trainees?**  **How would an geriatrics curriculum add value to your residency education?**  **How could orthopaedic residents benefit from greater exposure to geriatric medicine?**  *After describing the rotation:*  What would you add?  What do you feel is not necessary? |
| **Role of the Orthopaedic Surgeon** | What role do you see orthopaedic surgeons/residents in geriatric medicine outside of the OR?   - Is there room for improvement, and how should this be achieved? |

**Interview Questionnaire for Key Informants**

| **THEMES** | **Key Informants**  *(Orthopaedic Surgery Program Director, Geriatric Medicine Postgraduate Program Director, Orthopaedic Surgeons, Geriatricians, Charge nurses)* |
| --- | --- |
| **Effect of the Rotation** | **What changes, if any, do you perceive in the attitudes and/or practices of the older adult population in those residents who completed the orthogeriatrics rotation?**   - **What differences, if any, have you noticed regarding the knowledge, attitudes and skills of geriatric competencies between the junior and senior residents?**   **How comfortable do the residents feel in managing frail, older, complex patient? What differences, if any, exist between the junior and senior residents?** |
| **Exposure to Geriatrics** | To your knowledge, where else do the orthopaedic residents learn about geriatric medicine in your residency training?* |
| **Learning in the Rotation** | How important do you think a structured geriatrics rotation would be in helping trainees to manage perioperative issues (e.g. postoperative delirium, falls/frailty, pre-operative assessment, cognitive impairment, quality of life?)  **How would an geriatrics curriculum add value to your residency education?**  Within a 1 month geriatrics rotation, what do you think would be beneficial for orthopaedic residents to learn regarding perioperative care of the elderly? |
| **After describing the rotation** | What would you add?*  What do you feel is not necessary?*  After hearing the rotation described, do you feel that the structure/format is appropriate?* |
| **Role of the Orthopaedic Surgeon** | What role do you see orthopaedic surgeons/residents in geriatric medicine outside of the OR?   - Is there room for improvement, and how should this be achieved? |

****not applicable to Charge Nurses***
